# Evaluating the Impact of Intensive Case Management for Severe Vocational Injuries on Work Incapacity and Costs

**DOI:** 10.1007/s10926-021-09967-6

**Published:** 2021-03-11

**Authors:** Rolando Leiva, Lise Rochaix, Noémie Kiefer, Jean-Claude K. Dupont

**Affiliations:** 1 Hospinnomics (PSE – École d’Économie de Paris, Assistance Publique Hôpitaux de Paris – AP-HP) and UCL, London, UK; 2 Hospinnomics (PSE – École d’Économie de Paris, Assistance Publique Hôpitaux de Paris – AP-HP), Paris, France

**Keywords:** Case management, Disability leave, Matched case–control studies, Occupational injuries, Return to work

## Abstract

*Purpose* This study investigates the impact of an intensive case management program on sick leave days, permanent work incapacity levels and treatment costs for severe vocational injuries set up by the French National Insurance Fund in five health insurance districts. *Methods* The method employed relies on a four-step matching procedure combining Coarsened Exact Matching and Propensity Score Matching, based on an original administrative dataset. Average Treatment effects on the Treated were estimated using a parametric model with a large set of covariates. *Results* After one-year follow-up, workers in the treatment group had higher sickness absence rates, with 22 extra days, and the program led to 2.7 (95% CI 2.3–3.1) times more diagnoses of permanent work incapacity in the treatment group. With an estimated yearly operational cost of 2,722 € per treated worker, the average total extra treatment cost was 4,569 € for treated workers, which corresponds to a cost increase of 29.2% for the insurance fund. *Conclusions* The higher costs found for the treatment group are mainly due to longer sick leave duration for the moderate severity group, implying higher cash transfers in the form of one-off indemnities. Even though workers in the treated group have more diagnoses of permanent work incapacity, the difference of severity between groups is small. Our results on longer sick leave duration are partly to be explained by interactions between the case managers and the occupational physicians that encouraged patients to stay longer off-work for better recovery, despite the higher costs that this represented for the insurance fund and the well-documented adverse side effects of longer periods off-work.

**Supplementary Information:**

The online version contains supplementary material available at 10.1007/s10926-021-09967-6.

## Introduction

While case management has been practiced as early as the 1860s in the United States [1], it is relatively new in healthcare in France, with the first large scale case management program introduced in 2007 in an experiment targeting frail elderly autonomy enhancement through service coordination [2]. In the rehabilitation literature, case management is perceived as a tool to support rehabilitation after work injuries [3]. The process of rehabilitation and return to work (RTW) is complex and involves the work and coordination of many parties who provide support to the injured worker, such as relatives, insurers, employers, and care providers [4].

A prompt recovery of the injured worker is in the best interest of all stakeholders. From the employer’s perspective, a fast RTW may reduce some of the negative impacts of work disability, i.e. a lower individual and collective productivity (as disability imposes changes in the internal organization of labor), increases in insurance premiums as well as compensation costs [3, 5]. From the insurer’s perspective, better health outcomes could also result in lower treatment costs [1, 3] as well as reduced cash transfers (indemnities and pensions). Finally, fast RTW also entails societal gains in terms of lower transfers and a lower financial burden for welfare programs, overall increases in productivity and in the revenue base of social programs [5, 6]. From the worker and his household’s perspective, fast RTW will reduce health and quality of life losses, as well as income losses [5].

At the individual level, there is an important shift in focus from the pure biological aspects of RTW, mainly rehabilitation and restoration of functions, to a more holistic approach, known as the biopsychosocial model. This is mainly due to the interdisciplinary nature of disability. In a systematic review, Eggert [7] has identified four themes of psychosocial aspects to RTW: frustration, depression, discrimination, and obstacles in navigating complex workers’ compensation systems. Frustration stems from injured workers having to change roles at work and/or in the community, following the injury. At work it is often due to a lowering of expectations or to the fact that the new opportunity is not closely related to previous experience [7]. At home they may no longer be the breadwinner and may feel that they impose a burden on their community [7]. Depression has been shown to negatively impact workers’ capacity to RTW, which may be a consequence of stress associated with the injury and having to deal with complex compensation systems. Furthermore, discrimination against injured workers at the workplace further complicates the RTW process. Injured workers experience lack of respect and support while navigating the workers’ compensation system. They often report being misunderstood and unfairly treated, while facing hostility from co-workers and termination threats by employers. The system is often seen as rigid with disregards to individuals’ circumstances. The objective of a case-management coordinator would be to coordinate a treatment plan that addresses all of these aspects and considers the participant through a biopsychosocial lens.

Recent literature reviews have shown mixed results for disability interventions on outcomes such as RTW or cumulative sickness absence [8, 9]. Results have been shown to depend on program characteristics, intervention span [10], and heterogeneity within the treated group and related case management intensity [4]. An important contribution to this literature has recently been made by Scholz et al. [4] for the Swiss Health Insurance (Suva), with a 6-year follow-up randomized controlled trial (RCT) that compared two case management programs of different intensity levels. No significant effect was found on work incapacity, but treatment costs were found to be higher for the more intensive case management program, compared to the less intensive one.

The rehabilitation framework for injury at work in France has typically focused on the relationship between the injured worker and the occupational physician (OP), with little emphasis on coordination between all stakeholders. According to Belin et al. [6], France has “*a well-developed framework for rehabilitation and return to work, but coordination across the different steps of the return-to work process, from medical and vocational rehabilitation to reintegration at the workplace, is limited. As a result, return to-work considerations are generally dealt with only at the end of the sickness absence, with limited room for early intervention*” (p. 26). In France, the OP plays a crucial role for the rehabilitation process. For instance, the re-instatement to work is coordinated by the OP and the employer and, contrary to other European countries, it is the OP who decides whether the worker is able to return to his work and the tasks that can be performed [6]. It is also the OP who diagnoses the level of permanent incapacity, and therefore the level of compensation received by the worker. As a matter of fact, only 2% of vocational injuries lead to a severe permanent incapacity, but they represent 40% of vocational injuries’ costs for the National Health Insurance Fund. This high economic burden of permanent incapacity is consistent with the evidence from other high-income countries. For instance, a study on the costs of compensation for work-related injuries in sawmills in British Columbia found that the most expensive cost category was long-term disability, amounting to half of the total costs [11]. Another study for occupational injuries and illnesses in the United States in 2007 found that only 6% of the injuries led to permanent partial disabilities, but they represented 55.3% of the total medical costs [12].

In this context, and before the publication of the Swiss results, the French National Health Insurance Fund for Employees (CNAMTS) developed in 2014 an experimental program of intensive case management (ICM, hereafter) for workers with severe vocational injuries which hinder or severely delay RTW and generate the highest costs. Care coordinators were expected to develop a holistic, personalized treatment plan to support the injured worker through rehabilitation and ensure return to their previous job or a suitable alternative. Overall, the program’s aims were threefold: (1) restore the employees’ capacity after a work accident to the best of their ability, reducing physical, psychological, and relational post-trauma effects; (2) promote the professional and social reintegration of injured workers; (3) seek to improve the efficiency of the management of work-related injuries. Overall, based on the description of the case manager’s tasks and of the program’s objectives, it appears that the French ICM program reached beyond the sole biological dimensions of rehabilitation to a more holistic approach which includes some of the psychosocial aspects of RTW.

As in the Swiss ICM program, each case manager was in charge of defining a personalized rehabilitation plan for a maximum of 40 individually assigned injured workers (35 in the Swiss case). The program included home visits (at least once to enroll the beneficiary) and assistance with injury-related tasks (administrative, medical, social, professional). Both programs focused on severe accidents and had as objectives to support patients’ rehabilitation and to reduce costs. A RCT was initially considered but deemed infeasible for both ethical and practical reasons. Thus, the evaluation resorted to non-experimental matching techniques. The empirical strategy relied on creating balanced groups using a four-step matching procedure combining Coarsened Exact Matching (CEM) and Propensity Score Matching (PSM), taking advantage of a rich dataset containing key variables influencing the probability of treatment and the outcome results, and enabling a thorough control for the initial severity of the injury faced by the worker.

Our study aims at measuring the effects of coordinated care on several health and cost outcomes (see Table 1, Section "Covariates and Outcome Variables"), with a 12-month follow-up time. This one-year follow-up period is standard in the rehabilitation literature. Franche et al. [9] report only 3 studies (out of 10), Meijer et al. [13] only 2 (out of 15), and Vogel et al. [8] 4 (out of 12) with follow-up periods beyond one year. To the best of our knowledge, this is the first study to assess the impact of the French ICM program. Our results are similar to those of Scholz et al. [4] and add to the existing literature by adapting methods for program impact evaluations where randomization is not possible. Furthermore, our study presents two important differences from Scholz et al. [4]: (1) we study the sole effect of case management for occupational accidents (while the study by Scholz et al. includes non-occupational accidents of employed individuals and accidents of unemployed individuals) and (2) we compare intensive case management versus no case management while Scholz et al. compare two treatment intensities (intensive case management versus standard case management).

**Table 1:** Outcome variables

No.	Variable^a^	Description
Primary outcome variable		
1	Vocational sick leave days	Number of compensated sick leave days due to vocational injury
Secondary outcome variables		
2	Non-vocational sick leave days	Number of compensated sick leave days due to other health risks
3	Part-time RTW	Number of part-time compensated sick leave days
4	Workers with final medical certificate	Dummy variable with 1 if a final medical certificate (indicating recovery or consolidation of work incapacity status) is obtained in the 12-months follow-up period, and 0 otherwise
5	Workers with a permanent work incapacity (both moderate and severe)	Dummy variable with 1 for IP > 0 twelve months after the vocational injury
6	Workers with a severe permanent work incapacity	Dummy variable with 1 for IP > 9 twelve months after the vocational injury
7	Level of permanent work incapacity	Level of permanent work incapacity (IP) given by the occupational physician (0 if no IP given after 12 months)
8	Daily allowances for vocational sick leave	Total amount of vocational sick leave compensation
9	One-off indemnities	Total amount of indemnities for workers with 0 < IP ≤ 9
10	Life-long disability pensions	Total amount of pensions for workers with IP > 9
11	Healthcare costs for vocational injuries	Total amount of vocational-related healthcare costs, excluding hospital and emergency services
12	Healthcare costs for non-vocational sickness	Total amount of non-vocational-related healthcare costs, excluding hospital and emergency services
13	Total benefits	Sum of all cash and kind benefits (healthcare costs, daily allowances, disability pensions…), excluding hospital and emergency services

^a^All outcomes variables are measured over the year following the accident

## Methods

### Study Intervention

The ICM program was launched in November 2014 in five health insurance districts (CPAM), located in three different regions in France in order to cover a variety of geographical areas. In each district, there were one or more case managers allocated to the program and one part-time OP.

A subset of injuries was explicitly targeted by the ICM program, based on clinical expertise from a group of CNAMTS OPs: fractures, dislocations, sprains, profound wounds or amputations, located on limbs or trunk. The first task of the case manager was to identify eligible beneficiaries who had experienced vocational injuries (covering both work and work-commuting injuries). Eligibility was assessed based on the initial medical certificate that describes the type of injury and the initial sick-leave prescription. Although computer-assisted, the selection of injuries entailed large file processing by the case manager, with reference to eligibility flowcharts, and occasional advice from the OP. This complex and time-consuming screening process had to be carried out with limited information on severity. Each injured worker deemed eligible had to be visited by the case manager to initiate program participation.

For those who were eligible and enrolled in the program, the case manager developed a personalized rehabilitation plan. The case manager ensured that administrative files were filled-in, helped arrange health and social care professionals’ appointments and facilitate RTW. Their main role was to coordinate stakeholders: the injured patient, the employer, the national health insurer as well as health and social care professionals. Each case manager had a list of up to 40 cases.

### Inclusion/Exclusion Criteria

The data were extracted from the French National Health Insurance Database (SNIIRAM) and the linked national hospital discharge database (PMSI). The case managers’ database, used for the selection of eligible patients, was linked to the national database to identify beneficiaries. All data cleaning and analyses were performed using R Statistical Software, version 3.4.1. Ethical approval for the program and for this study was obtained from CNAMTS (reference numbers: 254-5-2014 and 46-29-2016).

The treated group was defined as all workers who enrolled by signing the letter of participation to the case management program and whose accident occurred in 2015. Accidents occurring in 2014 were not considered in the analysis because the program was still in its early days. Exclusion criteria were defined as follows: injuries that could not be identified in the national database due to coding errors; patients with chronic low back pain—who were offered a significantly different version of the case management program—; relapses, as they would not be comparable to initial injuries, either because the relapse variable indicated so or because workers received disability pensions during the first month after the accident. After implementation of these exclusion criteria, the treated group on which matching was performed contained 269 observations (see Section "Matching and Balance of Samples").

A first definition of the control group entailed all vocational injuries from the same five districts and the same year (2015) in which the program was implemented. However, as many as 79 treated individuals could not be matched by a control observation, resulting in an unsatisfactory balance between treated and control groups (see Online Appendix). The control group was subsequently extended to include vocational injuries occurring in both 2013 or 2015 in ten additional health insurance districts, chosen on the following criteria: a comparable population, both in demographic and socio-economic terms, comparable population frequencies of permanent disability levels after a work injury, and comparable administrative management methods. No significant changes occurred between 2013 and 2015 in the rehabilitation of injured workers in France. Some observations with no equivalent in the treatment group were excluded from the sample: (1) those with lesion codes outside the program’s target; (2) those with non-null values on disability pensions for this accident, sick-leave days and benefits for partial work or non-vocational sickness in the first month, which were always null for the treated.

This new control group contained 304,689 observations for matching implementation. By increasing the probability of finding individuals comparable to those who committed to the case management program, it substantially improved the balance between the treated and the control groups (see  Tables OA.2 and OA.3 in the Online Appendix).

**Table 2:** Average treatment effects on the treated (ATT) after matching

No.	Outcome variable	Control group (weighted)^a^	Treatment group (weighted)^a^	ATT [confidence intervals]^b^	Standard error	p-value	Ratio treated/control group [confidence intervals]^c^
1	Vocational sick leave days	237.461	259.275	22.025*** [13.110–30.940]	4.548	< 0.001	1.0 [1.0–1.1]
2	Non-vocational sick leave days	7.868	3.558	− 4.331* [− 9.202–0.541]	2.485	0.081	0.4 [− 0.2–1.0]
3	Part-time RTW	9.18	13.092	4.389* [− 0.270–9.048]	2.377	0.065	1.4 [0.9–1.9]
4	Workers with final medical certificate	0.445	0.475	0.023 [− 0.025–0.071]	0.024	0.349	1.0 [0.9–1.1]
5	Workers with a permanent work incapacity (IP > 0)	0.082	0.229	0.141*** [0.108–0.173]	0.017	< 0.001	2.7 [2.3–3.1]
6	Workers with a severe permanent work incapacity (IP > 9)	0.006	0.025	0.018*** [0.008–0.028]	0.005	< 0.001	4 [2.3–5.6]
7	Level of permanent work incapacity	0.473	1.4	0.881*** [0.654–1.108]	0.116	<0.001	2.8 [2.3–3.3]
8	Daily allowances for vocational sick leave (in euros)	11,003.42	12,475.37	1,194.46*** [622.247–1766.672]	291.925	< 0.001	1.1 [1.0–1.1]
9	One-off indemnities (in euros)	193.209	514.681	310.295*** [229.092–391.498]	41.427	< 0.001	2.6 [2.1–3.0]
10	Life-long disability pensions (in euros)	31.745	88.57	49.615*** [14.674–84.556]	17.826	0.005	2.5 [1.4–3.6]
11	Healthcare costs for vocational injuries (in euros)	3,118.93	3,673.47	353.039* [− 21.329–727.408]	190.991	0.065	1.1 [0.9–1.2]
12	Healthcare costs for non-vocational sickness (in euros)	1,129.76	1,160.93	− 2.673 [− 272.101–266.756]	137.454	0.984	0.9 [0.7–1.2]
13	Total benefits (in euros)	15,649.99	18,027.85	1,846.861*** [1,052.287–2,641.436]	405.367	<0.001	1.1 [1.0–1.1]

^a^Sample sizes: control group N = 240 and treatment group N = 13,567

^b^Calculated using a linear regression model in the matched sample using all the covariates as controls. Significance of coefficients indicated by *p-value 10% level, **p-value 5% level and ***p-value 1% level

^c^Ratio calculated using the ATT, as the sum of the control group value (1) + ATT value (2), divided by the control group value (1): (1 + 2)/(1). Confidence intervals calculated as the sum of the control group value (1) + lower (upper) bound of ATT’s confidence interval (2) divided by the control group value (1): (1 + 2)/(1)

### Covariates and Outcome Variables

#### Control Variables

Following Caliendo and Kopeinig [14], the control variables were chosen based on their availability in the national database and their likeliness to influence the probability of treatment or the outcomes. We divided covariates into three groups as documented in Table OA.1 in the Online Appendix.

The first group of variables includes a rich set of socio-demographic variables (such as gender, age, place of residence), and the worker’s record of past vocational injuries, the worker’s occupation, and their work environment.

For the second group, a number of variables were extracted directly from the initial medical certificate issued after the accident in order to provide a direct measure of the injury’s initial severity: the type of injury (categories: imprecise, superficial injuries, open wounds, closed fractures, open fractures, dislocations, sprains and strains, and traumatic amputations), the location of the lesion (categories: imprecise, neck, back and rib cage, upper limbs, lower limbs), the number and type of injury codes present in the initial medical certificate, and the initial number of sick leave days prescribed.

The third group of covariates provides an indirect measure of the injury’s initial severity: health care utilization, measured by the actual utilization of health care and the associated costs in the first month of the program. The assumption here, based on the extant literature (see [15, 16]), is that workers with more severe injuries are entitled to more sick leave days and need more care of a more expensive type. Among those covariates, we have healthcare services used (for instance, whether the patient was hospitalized during the first month, the number of hospitalizations, the number of medical or surgical procedures for the first hospital stay) and cost variables (such as health care reimbursement for consultations with general practitioners, physiotherapists, or nurse care). Since enrolling workers in the program took on average 1 month, the program should not have affected these variables during this first month.

#### Primary Outcome Measure

All outcome measures are presented in Table 1 with corresponding numbers. The primary outcome measure is the total number of compensated sick leave days (either full-time or part-time) at a given reporting date, i.e., at the end of 12 months after the vocational injury (variable 1). This cumulative measure is more informative than a one-time measure (like time to first RTW) because it includes part-time absences and possible recurrences of sick leaves [17, 18].

#### Secondary Outcome Measures

The number of sick leave days for non-vocational injuries (variable 2) is useful to check possible spillover effects of the ICM program, leading to a decrease in the number of sick leave days due to non-vocational related health risks. The number of days spent in part-time RTW (variable 3) allows to qualify the nature of RTW. A final medical certificate was issued by an OP and indicated either recovery or consolidation of work incapacity status. Comparing the number of workers with or without a final medical certificate at the end of the follow-up period is useful to assess the speed at which cases were handled (variable 4).

Other outcome variables relate to permanent work incapacity (IP hereafter, “*Incapacité Permanente*” in French). The IP grade is determined by the OP, based on the degree of remaining sequelae—both physical and mental—after rehabilitation. A rate between 1 and 9% is considered a *moderate permanent work incapacity* and entitles the worker to a one-off indemnity, the level of which increases with the IP grade. A rate above 9% is considered a *severe permanent work incapacity*, with a life-time entitlement to a disability pension. In France, an injured worker cannot receive both a one-off indemnity and a disability pension. In our study, we used two IP measures: one defined as the percentage of workers with IP > 0 (variable 5), and another focusing on workers with IP > 9, (variable 6). We also computed the average rate of IP (variable 7) to compare the extent to which treated and non-treated individuals differ in the level of IP as assessed by their OP.

The next three outcome measures relate to vocational injuries’ cash benefits: daily allowances for vocational sick leave (variable 8), one-off indemnities (variable 9), and life-long disability pensions (variable 10). Two outcome measures focus on benefits in kind, i.e., in relation to healthcare consumption (excluding hospitalizations and emergency services’ use, this information being unavailable), either due to the injury (variable 11) or not (variable 12). Total benefits (variable 13) are the sum of all in cash and in kind benefits paid by the insurer.

### Empirical Strategy

Combining a Difference in Differences (DiD) approach with matching is a commonly used strategy in the econometrics literature for similar cases, but it could not be implemented here due to data availability and sample size. Using a DiD approach would have called for an aggregate analysis carried out at the level of the insurance districts. We would therefore have had to compare treated districts with similar untreated districts. To do this, we would have had to define which untreated districts were similar to those treated ones, which could easily be done since for matching, we have included individuals from 10 other comparable health insurance districts. But limited sample size (269 treated observations in the five treated insurance districts), compared to a very large number of untreated observations (both in untreated and treated districts) would have made it difficult to see the effects of the program at this aggregate level. In statistical terms, the limited sample size for each treated district would make them appear at aggregate level like any untreated district. Also, the data only covers 2 years which does not allow a robust test of the parallel trends assumption. As a result, we chose a recent and elaborated matching approach which substantially improves the balance between the treated and control groups.

#### Matching Techniques

To ensure group comparability, we relied on a matching strategy to construct a control group using administrative data. Several methods have been proposed in the literature to define control groups. Exact matching on selected covariates and propensity score matching methods [14, 19] are the most commonly used. These methods are widely used in economics and social sciences, and have been applied to rehabilitation [20–22]. Our variable of interest is the Average Treatment effect on the Treated (ATT). The key issue is to identify a group of individuals that are similar to the treated ones on a set of X covariates, but that did not receive the treatment. In so doing, the differences found on outcomes Y between groups will be attributable to the treatment itself.

A crucial step for the reliability of the results is checking the balance of the covariates between treatment and control groups after matching. The objective is to create balanced groups based on a matching algorithm. The literature suggests trying several model specifications until a balance is reached between groups on their covariates [19]. Thus, testing the covariates balance is an important step in matching techniques. The distribution of the covariates between samples must be similar. Several methods are available to test balance quality, such as graphical methods, t-test of means’ differences between groups or re-estimating the propensity score among the matched sample [14]. The most commonly used technique relies on the “standardized bias” or the “standardized difference in means” for each covariate, computed as the difference of group means, divided by the standard deviation of the treated group pre-matching [19, 23]: $(\overline{X}_{{\text{T}}} - \overline{X}_{{\text{c}}}) /\sigma_{{{\text{Tpre}}}}$.

Thus, after each matching attempt, we assessed covariate balance on the matched samples. The difference between groups was considered significant if the absolute value of the standardized bias was above 0.1 [24–26]. We used the cobalt package version 3.3.0 [23] to compute these, as well as variance ratios for each continuous covariate. Several authors [25, 27] encourage the use of this measure which gives a numerical diagnostic on the second-order of covariate distributions. Ratios close to 1 indicate similar variance in the treatment and the control groups. The difference between groups was considered significant if the ratio—defined in cobalt such that the numerator is the greatest variance—was above 2 [19, 28, 29]. For the initial prescription of sick leave days, one of the variables deemed the most predictive of the main outcome variables, we used graphical comparisons of the group distributions [23, 25, 27, 30], using the package ggplot2 version 3.3.0 [31].

Even after trying different designs and model specifications (as the number of neighbors, caliper size, allowing for replacement of matched controls or not), the quality of matching (measured in the covariate balance assessment) remained insufficient, especially for some important variables with non-normal distributions (multiple peaks or highly skewed).

To further improve on matching quality, we chose to follow the combined matching methods adopted in Jones et al. [32, 33]. It uses a preprocessing (see Ho et al. [27]) of the data with Coarsened Exact Matching (CEM), combined either with Propensity Score Matching (PSM) or Entropy Balancing. CEM allows to perform exact matching on variables that are coarsened, i.e. they are recoded in such a way that substantively indistinguishable values are grouped into the same category [34]. CEM circumvents the rigidity that exact matching imposes (i.e., first transforming these covariates into categorical variables and then performing exact matching) and compared to simple PSM, it has the advantage of giving a better control on the balance of covariates deemed most relevant [32].

#### Implementing Combined Matching

Four sequential steps were followed to implement this combined matching procedure:

- Step 1. Preprocessing of the data through CEM with important covariates
  We chose a set of covariates on which a tight match was desired but difficult to achieve: number of previous vocational injuries, previous levels of permanent disability, initial prescription of sick leave days, number of sick leave days during the first month after the accident, lesions’ type, lesions’ location, and having received hospital care in the month following the accident. These covariates were used to control for the initial severity and the worker’s history of vocational injuries. These variables and associated coarsening cut-offs were adjusted to minimize imbalance on the matched samples, at least for the variables included in the CEM, while minimizing loss of treated observations through lack of appropriate match. At the end of this first step, a preprocessed dataset was obtained with weights derived from CEM: unmatched observations were weighted 0, matched treated observations were weighted 1, and matched controls were weighted according to the number of matched treated and control observations in the strata and overall. We used the R package CEM version 1.1.9 to compute our estimations.
- Step 2. Construction of a propensity score with all control variables
  A logistic regression predicting treatment status using all control variables was performed on the preprocessed sample. This propensity score was derived on the weighted data after the first CEM preprocessing, so that it could be focused on the determinants not accounted for by the first CEM. Propensity score values between the treated and the control groups were compared, and observations potentially dropped when out of the common support.
- Step 3. Processing of the data through CEM with important covariates and propensity score
  We coarsened the propensity score computed on the preprocessed sample. Several cut-offs were tested to obtain a good balance on all covariates while minimizing loss of treated sample. We applied CEM on the pre-processed sample (with weights derived from the first CEM), while including the important coarsened covariates and the coarsened propensity score. We then assessed the balance of the matched samples.
- Step 4. Estimation of the Average Treatment effect on the Treated
  Results were estimated on the processed data (with weights derived from the second CEM). We computed a parametric ATT by conducting linear regressions with all control variables. This method is recommended to capture any remaining residual effect of these control variables on outcomes [19, 27, 33, 34]. T-tests were performed to assess statistical significance, which was set at p ≤ 0.05.

## Results

Results are presented in two sections. Section "Matching and Balance of Samples" documents the performance of the combined CEM and PSM preprocessing at balancing covariates between the treatment and control groups, while Section "Outcome Results" presents the program results.

### Matching and Balance of Samples

The initial sample consisted of 269 treated individuals and 304,689 potential controls. It displayed considerable imbalance: 50 out of 63 standardized mean differences and 11 out of 18 variance ratios were beyond the chosen thresholds (see Table OA.2 in the Online Appendix).

In the first step of preprocessing with CEM, we coarsened the number of previous vocational injuries in four categories, previous levels of permanent disability in five categories, initial prescription of sick leave days in six categories, number of sick leave days during the first month after the accident in four categories, while lesions’ type, lesions’ location, and having received hospital care in the month following the accident were left uncoarsened. This created 3,828 strata, but only those 182 strata which contained both treated and control individuals were kept. The sample was thereby reduced to 260 treated individuals and 45,235 controls. The balance was improved with only 20 standardized mean differences and one variance ratio remaining imbalanced.

**Fig. 1:**
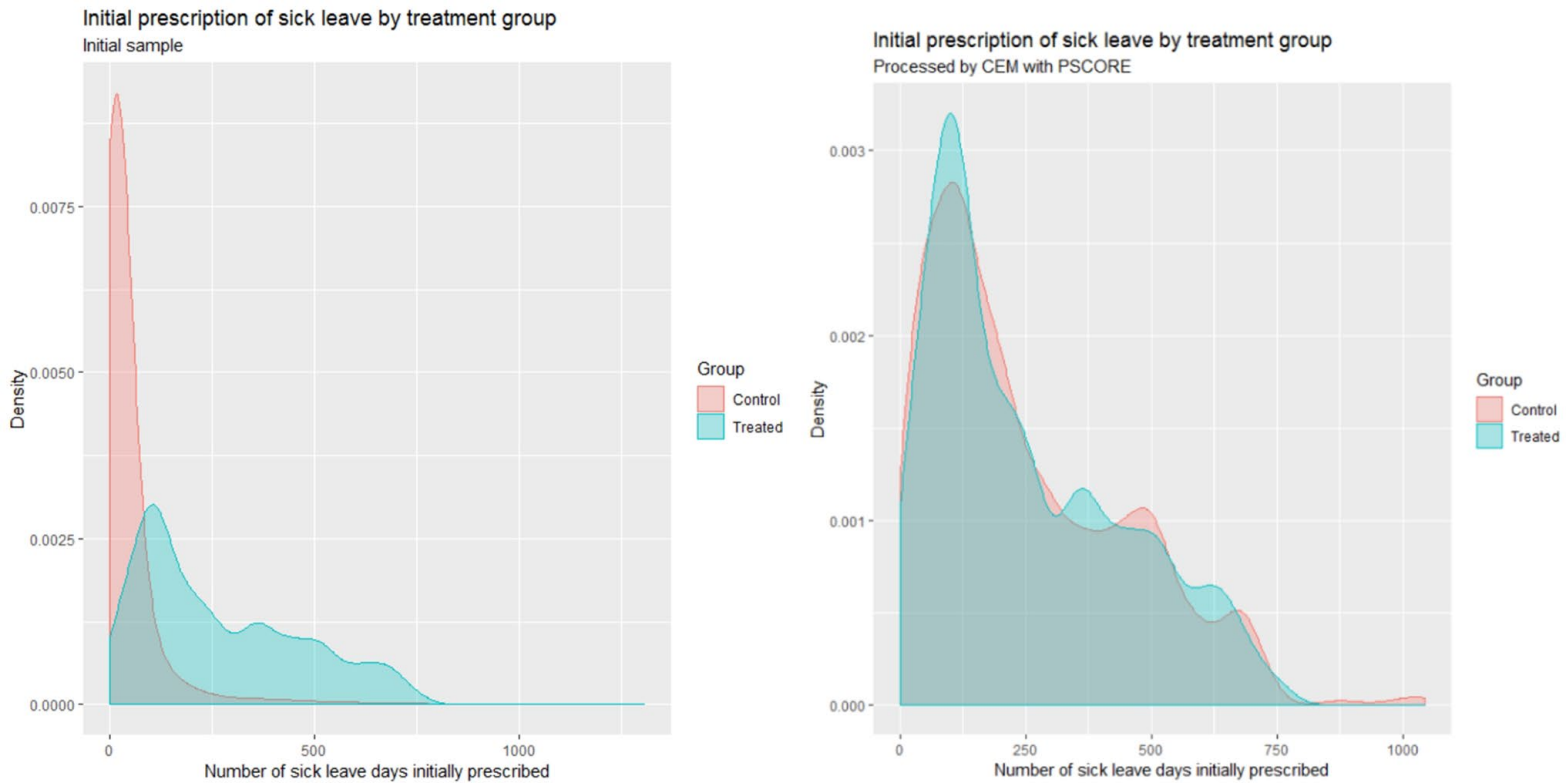
Initial prescription of sick leave by treatment group, before and after matching

For the second CEM (step 3) including the propensity score (calculated in step 2), the best balancing results were found by coarsening the propensity score into quintiles (computed on the weighted data pre-processed by CEM). This created 835 strata, yet only 206 of them with matching treated and controls were retained to make up the final sample. For the analysis of outcomes, our matched sample is composed of 240 treated individuals and 13,567 control individuals. Balance is obtained on all control variables with the exception of urban area beyond 200,000 inhabitants which is very slightly above the threshold (standardized bias equal to 0.101, in Table OA.2 in the Online Appendix). The balance improvement due to using the combined matching strategy can be assessed visually on the density plots in Fig. 1, for one of the most predictive variables: initial prescription of sick leave days. Although 29 treated observations were lost in the process, the final balance is satisfactory. Except for Scholz et al [4], our sample size is greater than that found in most of the studies identified in the literature reviews of Franche et al. [9], Meijer et al. [13] and Vogel et al. [8]. Franche et al. for instance, report 5 studies (out of 10), Meijer et al. 16 (out of 22) and Vogel et al. 8 (out of 14) with fewer than 100 treated individuals.

### Outcome Results

Table 2 reports the full set of results for primary and secondary outcome variables at the end of the one-year follow-up.

#### Vocational Sick Leave Days

The number of sick leave days due to a vocational injury during the first year after the accident is on average higher in the treated group (259 days) than in the control group (237 days). We obtained an ATT of 22.0 extra sick leave days for a treated worker (95% CI 13.1–30.9). This effect is highly significant (p-value < 0.001).

#### Non-Vocational Sick Leave Days

A plausible explanation for the increase in the number of vocational sick leave days could be an unexpected spillover effect of the ICM program, potentially leading to a decrease in non-vocational sick leave days. In cases where workers have both vocational and non-vocational related illnesses, one would expect a single claim to be filed to the vocational risk fund, and more so for the treatment group if it is done by the case manager. We find that a treated worker has on average 4.3 days less in non-vocational sick leave compared to a worker in the control group. However, this result is not significant at the 5% (p-value = 0.081) but at the 10% threshold. This result shows that it is important to control for potential spillover effects from vocational to non-vocational health risks, particularly when they are covered by separate insurers, as is the case in most countries but France.

#### Part-Time Return to Work

These additional vocational sick leave days for a treated worker could also be partly due to an increase in the number of days spent in part-time RTW. Yet we only find 4.4 extra days in part-time out of 22, but with a p-value of 0.065, i.e., slightly above the significance threshold.

#### Final Medical Certificates

A final medical certificate is issued by the OP when the worker’s health status is stabilized, stating whether the worker presents a permanent work incapacity, and the level of this incapacity. Our results show that the program did not have a statistically significant impact on the proportion of workers who obtained a final medical certificate one year after their accident (44.5% in the control group and 47.5% in the treatment group, p-value 0.349).

#### Permanent Work Incapacity

The proportion of workers who received a permanent work incapacity (both moderate and severe, IP > 0) one year after the accident was greater in the treatment group (22.9%) than in the control group (8.2%). The ATT indicates that an additional 14.1% (p-value < 0.001) of treated workers received a strictly positive IP. Compared to the control group, this means that, one year after the accident, the program led to 2.7 (95% CI 2.3–3.1) times more permanent work incapacity in the treatment group.

This higher level of permanent work incapacity in the treated group cannot be explained by a different timing in assessment of sequelae since, as mentioned previously, the proportion of workers with a final medical certificate is similar in both groups. Neither should differences in the initial severity level be driving this result as the matching strategy adopted has achieved a good balance of initial severity between groups. It seems also highly unlikely that the program, by providing more care, would have led to worse physical outcomes.

To further document this extra 14.1% of permanent work incapacity for the treated group, we use the second outcome variable for permanent work incapacity that separates out the more severe from the moderate permanent vocational incapacities (IP > 9, variable 6). We find only an additional 1.8% (p-value < 0.001) of treated workers receiving a severe permanent incapacity. We therefore conclude that most of the increase must have come from the moderate work incapacity levels (0 < IP ≤ 9).

We also documented to what extent the work incapacity severity differs by groups by computing the average degree of permanent work incapacity (variable 7). We found an average degree of 0.473 in the control group and 1.4 in the treated group, with an ATT of 0.881 (p-value < 0.001). Thus, we found that even though the treated group presents a higher proportion of workers with an IP>0, the average difference in the severity between groups is less that one percentage point, which is very low. For example, a rate of permanent incapacity of 1% and 2% entitles the worker to a one-off indemnity of 410.71 and 667.54 euros, respectively. Hence, we conclude that workers in the treated group do not present significantly lower health outcomes than those in the control group.

#### Cash Benefits and Treatment Costs

The increase in both sick leave days and permanent work incapacity rates for treated workers automatically leads to higher compensation costs. Over the first year, on average:

- Daily allowances for vocational sick leave (variable 8) are 1,194 € higher for a treated worker (p-value < 0.001).
- One-off disability indemnities for permanent work incapacity of level 1 to 9 (variable 9) are 310 € higher for a treated worker (p-value < 0.001). This suggests that for a comparable severity level in the first month, treated workers received a higher IP assessment and therefore a higher compensation.
- Life-long disability pensions for severe permanent work incapacity (variable 10) are 49 € higher for a treated worker (p-value 0.005).

In addition, healthcare costs related to the vocational accident (healthcare benefits in kind) are also higher for treated workers, although not statistically significant (353 €, p-value 0.065). This additional care does not seem to originate from spillover effects (from vocational illnesses to non-vocational ones), because the ATT on the latter variable is highly non-significant (p-value 0.984).

All of these additional costs for the treated group add-up to higher total healthcare benefits distributed by the insurance fund over the year following the accident: a treated worker costed on average 1,847 € (p-value < 0.001, 95% CI 1,052–2,641 €) more than one from the control group, equivalent to an additional 11.8%. Once we add the yearly operational costs of 2,722 € per treated worker, we obtain an average total additional treatment cost of 4,569 € per treated worker (95% CI 3,774–5,363 €), corresponding to a cost increase of 29.2% for the insurance fund.

## Discussion

Contrary to expectations, we have found that workers in the ICM program spent 22 more days in sick leave than those in usual care. Compared to existing results, one study [18] finds a higher number of sick leave days for the treated group, but the comparison is made with a light intervention, not usual care, as is the case here. In most other studies, case management either reduced sick leave time or time to RTW [9, 35–37], or had no significant effect [4, 13, 38, 39]. However, these studies often include broader categories of injuries than just vocational accidents and use different measures, making comparisons difficult. Compared to Scholz et al. [4], for instance, our primary outcome variable is measured over 12 months (compared to 6 years) after the accident, and our study focuses solely on vocational injuries. Our primary outcome measure also differs from theirs, which relies on a percentage reduction in work-capacity.

We have also found that the proportion of workers with permanent work incapacity (workers with an IP > 0) is considerably higher in the treatment group than in the control group (22.9% versus 8.2%, p value < 0.001). This result is driven by the moderate work incapacity category (0 < IP ≤ 9), because only 1.8% more workers in the ICM program received a severe permanent work incapacity (IP > 9), compared to usual care. These results contrast with findings from the related literature evaluating similar interventions that either found improvements [40] or no effects on permanent work incapacity [38].

However, our results on the prevalence of moderate work incapacity are close to those from Scholz et al. [4]. In their study, they found that there is no significant difference between the proportion of workers receiving a disability pension, but the proportion of patients receiving integrity indemnities was higher in the Intensive Case Management intervention (37%) than in the Standard Case Management (32%). These indemnities are one-off amounts paid to those workers suffering permanent damage to their physical or mental integrity, similar to the one-off indemnities received by workers in France in the moderate work incapacity category. Indeed, and as presented previously, most of the total effect on permanent work incapacity in France comes from this category. Thus, as in Scholz et al. [4], we found that a higher proportion of workers treated in the Intensive Case Management program have *permanent but moderate health sequelae*.

Yet, this result does not necessarily imply that workers treated in the ICM program have significantly lower health outcomes. We have computed the average permanent work incapacity rate between groups, and we found it to be 0.473 in the control group and 1.4 in the treatment group, with an ATT of 0.881 (p-value < 0.001). We therefore conclude that the program did not lead to significantly lower health outcomes. In the case of Scholz et al. [4], they found a higher but almost identical average degree of disability for patients receiving a pension in the ICM program compared to the SCM program (34.7 versus 34.9, with an ATT of 0.4 and a p-value of 0.90).

Moreover, only a little increase (not even significant at 5%) of part-time RTW was observed in the French ICM program. Similar results of very low to no-significance have been found in other studies [41, 42]. Our results are surprising considering that part-time RTW is actively encouraged in several EU countries [6], based on the existing empirical evidence showing that long sick leave spells may have negative effects (such as depression, social isolation and inactivity) [43, 44]. If, as has been suggested in the extant literature, part-time RTW increases the probability of full recovery [45], and contributes towards a faster recovery [46, 47], this result may partially explain why the program did not reduce the number of sick leave days nor the number of individuals receiving a diagnosis of permanent work incapacity.

As expected from those findings, the ICM program running costs were not compensated by decreases in cash or in kind benefits. While several previous studies found that case management lowered costs [9, 17, 37], only a few found that ICM programs increased costs [4, 48]. Scholz et al. [4] for their part, did find higher costs: + 9.4% for the more intensive program compared to the less intensive one. Our findings of a higher extra cost (+ 11.8%) may be explained by the fact that we compare an intensive management program to usual care, while Scholz et al. [4] compare two case management programs of varying intensity. Furthermore, while our positive and significant results are obtained for the 12-month-period after the injury, Scholz et al. [4] found that the cost difference is not significant in the first year of the intervention, but that it increases steadily over the remaining 5 years.

When discussing their non-significant results regarding RTW, which they find counter-intuitive, Scholz et al. [4] “*speculate that there may have been a tendency for case managers to prolong their efforts and ‘overcare’ for patients rather than to limit personal assistance to what is necessary under an economic maxim. This may also have been the consequence of a certain pressure for success felt by case managers*” (p. 326). This explanation also seems to apply in our case. Since case managers were mandated to ensure a sustainable RTW, with no instructions regarding costs, it is not surprising to find comparatively higher costs as case managers assumed that more care and more rest would lead to better RTW. Given that this extra care did not seem to reduce sick leave days or more generally, work incapacity, we tend to also conclude that the ICM program advised for more care and longer time off-work, beyond what would be expected both from a medical and a financial point of view, even when there was little chance for this extra care to bring significant improvements.

Admittedly, this practice of encouraging more off-work days is not congruent with the international rehabilitation practices and may in fact lead to worse health outcomes. There is empirical evidence of the benefits of work and re-employment after a period of sick leave [43]. Interventions aimed at a fast RTW are cited as part of the best practices for rehabilitation and are applied in the countries with the most comprehensive rehabilitation programs in Europe, which recognize the value of work for the recovery process [6]. In France, prolonged sick leave has been found to reduce the probability of being fit to return to the previous job (e.g., without any restrictions or modifications to be made in the workplace) [49]. Moreover, it seems that the value of work is not always recognized by all the stakeholders involved in the rehabilitation process [50]. For instance, according to Belin et al. [6], in France there are incentives for an early RTW from the patient’s perspective, but the financial incentives for the employers to reintegrate their workers are more limited and subject to the worker being recognized as disabled.

In addition, it seems that in France a biomedical vision of rehabilitation persists among some healthcare practitioners. Even though the more comprehensive biopsychosocial approach for rehabilitation is encouraged by health authorities [51] and evidence of its use in the management of some illnesses exists [52], it remains little understood and under-used. For instance, one qualitative study in the case of low back pain rehabilitation has found that some physicians have a biomedical vision of rehabilitation centered on the cure of the illness without caring for RTW [50]. Lack of coordination and collaboration between different professionals and employers (often justified by medical confidentiality), over-medicalization, and lack of human and financial resources for workplace interventions have been identified as barriers for a more integral process of rehabilitation based on a biopsychosocial approach [50, 52]. While the ICM program attempted to adopt a more holistic approach than what is usually done in France, the system did not necessarily empower case managers to follow this approach, despite the existing evidence and recommendations in the international literature.

Moreover, in the French case management program, the OP who evaluated a potential beneficiary at the onset of the program was also the medical reference for the worker’s case manager. The OP thus had a much deeper knowledge of a treated worker’s case than that of any other worker in the control group. This may have contributed towards making a more accurate severity assessment, which would then point to a systematic IP underrating in the standard process. Alternatively, a positive IP rate may have been chosen more often for the treated group out of compassion (since a positive IP rate leads to financial compensation). As a matter of fact, the scale used for the assessment of permanent work incapacity in France gives some latitude of choice to those in charge of grading severity. It is therefore likely that our results on sick leave duration are partly to be explained by the fact that the interactions between the case managers and the OPs have encouraged patients to stay longer off-work, for better recovery, and this despite the costs and adverse side effects associated with longer periods off-work, which have been well documented [43, 44].

Our study is the first impact evaluation in France of a case management program aimed at both improving physical rehabilitation and reducing time to RTW of injured employees, while also containing the health insurance costs associated with these injuries. An important strength of our study was the specific focus on vocational injuries, compared to previous research which did not introduce such a distinction between vocational and non-vocational risks. For instance, in Scholz et al. [4], vocational accidents only represented 37% of the total sample, the rest consisting of non-vocational injuries of employed workers, and a few injuries of unemployed people. Yet work-related injuries are associated with specific challenges in the rehabilitation process, linked to often poorer recovery outcomes [53], and a complex set of shared compensations between the employer, the State and the two different insurance risks (vocational and non-vocational) [6]. In our study, due to the rich dataset, we were able to focus on vocational injuries while controlling for potential compensation effects from vocational to non-vocational risks, in terms of healthcare use.

The empirical strategy implemented in this study shows the potential for quantitative analyses based on observational data, in the absence of a RCT. We take advantage of the large size of the available administrative dataset to find appropriate controls for the treated group. Our empirical strategy clearly remains a “second-best” option compared to a RCT, because it relies on the hypothesis of conditional independence which cannot be tested. But we are able to produce results through a combined CEM and PSM procedure, backed with careful balance checks.

Implementation issues, however, played an important role in determining the final size of the treatment group, the follow-up duration, and the final decision to redesign the program. For instance, the degree of latitude and the complexity of the eligibility criteria were associated with increased workloads for case managers and subsequent delays in entry, leading to a smaller than expected treated sample. Unexpected severity heterogeneity among beneficiaries was also a drawback of the program, often shared by others, such as the French evaluation of the frail elderly autonomy enhancement program [54]. Benefits in terms of reduced primary care or hospital care use could also potentially appear in the long-run, due to a higher quality rehabilitation provided in the program, but this could not be investigated within the study time frame. Finally, it was not possible to adopt a broader patient perspective, although previous studies have shown that patient satisfaction can be a highly relevant predictor of improved outcomes [55]. Precise information on workers’ quality of life and actual work status (either full-time or part-time RTW, unemployment or early retirement) at the end of sick leave indemnities was not available. While the program could have had positive impacts on these dimensions, they remain unaccounted for in this evaluation.

## Practical Implications for Future Case Management Programs’ Implementation

Based on the evaluation of this specific French ICM program, we cannot conclude on the general efficacy of case management programs. But the experience gained in evaluating the program, from its onset to its redesign, is worth sharing, with both policy and methodological recommendations for future comparable programs’ design and evaluation:

- First, one of the main threats to a program’s efficiency resides in the identification of eligible patients. Lack of appropriate data or recurring adjustments in the eligibility process, which are common in the early days of an intervention, can lead to increases in case managers’ workloads, subsequently delaying workers’ entry into the program. Making eligibility rules simple, with little room for interpretation by case managers is an important condition for success.
- Second, the program intensity, which directly affects outcomes [18, 56, 57], should be carefully tailored to the injured workers’ needs. In our study, the severity of the beneficiaries was lower than expected but the program intensity was not subsequently revised downwards, leading to care overuse and higher costs. Designing intensity-tiered case management programs is essential to cope with unexpected heterogeneity in selected beneficiaries’ severity levels.
- Third, training program implementers is crucial as inefficiency is more likely to arise in the early days. The presentation of the program’s main and secondary objectives is essential during these training sessions and it is important to recognize that framing effects may be present, just as they are in lab or field experiments. In our case, the choice made in the ICM program design to leave the secondary objective (reducing health insurance costs) implicit has had an impact on both OPs’ and case managers’ choices.
- Fourth, case management programs are more likely to be considered efficient if non-monetary outcomes—such as satisfaction, quality of life, and actual work status at the end of the compensated sick leave period—are accounted for in the benefits evaluation. Defining the right set of indicators at the onset of the program is essential for such a societal perspective to be adopted in the evaluation of the benefits. Moreover, a quantitative evaluation of a case management program should be complemented with a qualitative study to understand which factors are barriers and facilitators for reaching the program’s objectives. Analyzing the rehabilitation lens adopted by stakeholders (i.e., biomedical versus biopsychosocial), their degree of freedom within the general rehabilitation system and the individual, legal, and organizational barriers they face, would help not only to better understand the results obtained from the evaluation but also to determine to what extent they can relate to other countries’ experience.

## Conclusions

In line with the Swiss experiment evaluated by Scholz et al. [4], our results show that an ICM program is not sufficient to reduce work incapacity of severely injured patients. It can even increase time off-work and lead to upward revisions of the worker’s incapacity level. In our evaluation of the French ICM program, we found an increase in permanent work incapacity, which is not in line with the rest of the rehabilitation literature. However, the increase is mostly driven by an increase in moderate rather than severe work incapacity, and the difference in the average level of work incapacity between the treatment and the control group remains small.

We also find a higher percentage increase in total treatment costs than in the Swiss case, mainly due to the number of extra daily allowances paid for by the insurance fund. In our study, we therefore find that the implementation of an ICM program, comparable to the Swiss one, leads to even more unsustainable financial outcomes. This is partly due to the fact that our evaluation took place in the early stages of the program, when the balance was still being sought between the injured worker’s needs, in relation to severity, and the program’s response. We also hypothesize that case managers and OPs in the French ICM program may have tended to prescribe sick leaves more generously and, given their latitude of choice on the severity diagnosis and the fact that a positive IP rate leads to financial compensation, they may have granted their patients a slightly higher degree of permanent work incapacity out of compassion, thereby increasing the overall financial costs of the ICM program.

The combined matching strategy adopted in this study has proved useful for impact evaluations where RCTs cannot be implemented. In such cases, elaborate matching strategies become a powerful tool for constructing comparable treatment and control groups from observational data. Based on theoretical and previous empirical developments, we have illustrated the value of using this four-step method combining techniques such as propensity score, coarsened exact matching and preprocessing, which are useful when a single matching technique does not provide balanced groups.

Our study provided timely feedback to the French National Health Insurance Fund on the impact of the program. Indeed, it enabled an evidence-based decision to better tailor the original case management program to the needs of the target population, in relation to severity levels. While our study does not imply that intensive case management programs are not efficient for the rehabilitation of workers after a vocational injury in France, it led to the production of several policy recommendations that will prove useful for future programs’ design, implementation, and evaluation. For instance, this experiment has shown the existence of a learning curve for case managers, together with important behavioral responses on their part, which must be accounted for both in the program design and in the results. Also, the close experiment follow-up has demonstrated the need to carry out real time measures in order to identify potential program inadequacies as early as possible. It has also shown the need to prospectively define indicators that will adequately measure the program’s ability to meet its alleged objectives (such as improving time to RTW or reducing costs). Equally, developing a multi-level support program, according to different levels of intensity or adapting it to the specific environment of the injured worker will enhance efficiency in the use of limited resources. Finally, the French ICM program seems to indicate that if case managers are not given the tools to fully support injured workers, following rehabilitation recommendations derived from a biopsychosocial framework, they will tend to prescribe more sick day leaves and help injured employees obtain higher compensations, which in the end may work against improving their health outcomes.

## Supplementary Information

Below is the link to the electronic supplementary material.

Supplementary file1
