# Supplementary material for: Evaluating the Impact of Intensive Case Management for Severe Vocational Injuries on Work Incapacity and Costs
Source: J Occup Rehabil. 2021 Mar 11;31(4):807–21. doi: 10.1007/s10926-021-09967-6 (PMC8558282; doi:10.1007/s10926-021-09967-6)
Supplement: Supplementary file 1 — Supplementary file1 [file 10926_2021_9967_MOESM1_ESM.pdf]

Online Appendix

Table OA.1: Covariates used in the 4-step matching procedure and in the regression analysis for ATT calculation

**Covariates**		
**Group of variables**	**Variables**	**Presentation**
Sociodemographic, occupational and previous record of past vocational injuries variables	Worker's sociodemographic characteristics and place of residence	Sex, age, number of beneficiaries of his/her social security coverage, size and deprivation index of town of residence. Variables 1-12 in table OA.2
	Worker's record of past vocational injuries (before the studied accident)	Total previous permanent work incapacity and number of previous vocational injuries. Variables 13 and 14 in table OA.2
	Worker's occupation and work environment	Socio-professional category, type of employment contract, business sector of the firm, size of the firm. Variables 15-29 in table OA.2
Direct measures of worker's initial severity	Type of accident and injury	Type of vocational accident (in the workplace or on the way to work), type of injury, location of the injury, number of eligible injury codes detected in the initial medical certificate, absence of specific injury codes, and initial number of sick leave days prescribed. Variables 30-47 in table OA.2
Indirect measures of worker's initial severity	Variables of healthcare treatments and costs, measured during the first month after the accident	Number of sick leave days for vocational accident during the first month after the accident, total amount of daily allowances for sick leave for vocational accident during the month after the accident, and all the variables between 48 and 63 in table OA.2

Table OA.2: Descriptive statistics before and after matching

The values above the chosen thresholds of balance are indicated in bold [a/]

N°	Variable		Before matching: Mean – Control	Before matching: Mean – Treated	Before matching: Standardized bias	Before matching: Variance Ratio	After matching: Mean – Control	After matching: Mean – Treated	After matching: Standardized bias	After matching: Variance Ratio
	Sample size		304,689	269			13,567	240		
1	Sex: Male (Ref: Female)		0.608	0.710	**0.224**		0.691	0.700	0.020	
2	Age		37.789	42.297	**0.378**	1.034	41.672	42.104	0.036	1.010
3	Number of beneficiaries of his/her social security coverage		1.613	1.587	-0.023	1.110	1.560	1.575	0.014	1.197
4	Size of the hometown	Rural	0.110	0.108	-0.006		0.114	0.100	-0.046	
5		City up to 199,999 inhabitants	0.248	0.152	**-0.266**		0.165	0.150	-0.042	
6		City of more than 200,000 inhabitants	0.299	0.494	**0.391**		0.450	0.500	**0.101**	
7		Greater Paris area	0.344	0.245	**-0.228**		0.271	0.250	-0.048	
8	Deprivation index of the hometown	Most privileged quintile	0.241	0.264	0.052		0.245	0.258	0.030	
9		2nd quintile	0.208	0.279	**0.158**		0.295	0.283	-0.026	
10		3rd quintile	0.163	0.178	0.040		0.189	0.179	-0.025	
11		4th quintile	0.149	0.104	**-0.148**		0.109	0.104	-0.015	
12		Most deprived quintile	0.239	0.175	**-0.168**		0.162	0.175	0.036	
13	Total previous permanent work incapacity		0.461	1.074	**0.138**	**3.176**	0.458	0.563	0.033	1.638
14	Number of previous vocational accidents		1.570	1.688	0.056	1.080	1.505	1.508	0.002	1.032
15	Socio-professional category	Unspecified or miscellaneous	0.305	0.190	**-0.294**		0.183	0.183	0.001	
16		Managers, technicians	0.068	0.074	0.024		0.083	0.071	-0.045	
17		Employees	0.316	0.294	-0.049		0.330	0.321	-0.020	
18		Unskilled workers	0.128	0.141	0.038		0.114	0.138	0.069	

N°	Variable		Before matching				After matching			
			Mean		Standardized bias	Variance Ratio	Mean		Standardized bias	Variance Ratio
			Control	Treated			Control	Treated		
19		Skilled workers	0.183	0.301	**0.257**		0.290	0.288	-0.006	
20	Type of employment contract	Unknown or miscellaneous	0.351	0.238	**-0.264**		0.247	0.246	-0.004	
21		Permanent contract	0.544	0.651	**0.223**		0.649	0.638	-0.025	
22		Temporary contract	0.105	0.112	0.020		0.103	0.117	0.042	
23	Business sector of the firm	Commercial	0.345	0.342	-0.007		0.351	0.338	-0.029	
24		Industrial	0.210	0.305	**0.205**		0.274	0.296	0.048	
25		Services and miscellaneous	0.445	0.353	**-0.191**		0.375	0.367	-0.017	
26	Size of the firm	Microenterprise (9 or less)	0.176	0.286	**0.244**		0.295	0.283	-0.025	
27		Small and medium sized enterprise (10 to 249)	0.399	0.405	0.013		0.391	0.408	0.036	
28		Intermediate size enterprise (250 to 4,999)	0.285	0.208	**-0.188**		0.193	0.196	0.008	
29		Large company (5,000 or more)	0.141	0.100	**-0.134**		0.122	0.113	-0.032	
30	Type of vocational accident: In the workplace (Ref: On the way to work)		0.845	0.762	**-0.195**		0.770	0.746	-0.056	
31	Type of injury	Imprecise	0.600	0.223	**-0.903**		0.242	0.242	0.000	
32		Superficial injuries	0.066	0.019	**-0.351**		0.017	0.017	0.000	
33		Open wounds	0.079	0.052	**-0.121**		0.046	0.046	0.000	
34		Closed fractures	0.051	0.405	**0.720**		0.408	0.408	0.000	
35		Open fractures	0.008	0.119	**0.341**		0.100	0.100	0.000	
36		Dislocations, sprains and strains	0.194	0.130	**-0.191**		0.142	0.142	0.000	
37		Traumatic amputations	0.001	0.052	**0.228**		0.046	0.046	0.000	
38	Location of the injury	Imprecise	0.135	0.123	-0.039		0.117	0.117	0.000	
39		Neck, back and rib cage	0.283	0.130	**-0.454**		0.133	0.133	0.000	
40		Upper limbs	0.309	0.353	0.093		0.342	0.342	0.000	
41		Lower limbs	0.273	0.394	**0.248**		0.408	0.408	0.000	

N°	Variable	Before matching				After matching			
		Mean		Standardized bias	Variance Ratio	Mean		Standardized bias	Variance Ratio
		Control	Treated			Control	Treated		
42	Number of eligible injury codes detected in the initial medical certificate	1.162	2.487	**0.663**	**2.853**	2.325	2.508	0.092	1.135
43	Absence of low back pain code in the initial medical certificate (Ref: presence)	0.656	0.874	**0.655**		0.866	0.867	0.001	
44	Absence of amputation code in the initial medical certificate (Ref: presence)	0.998	0.944	**-0.233**		0.951	0.950	-0.004	
45	Absence of multiple fractures code in the initial medical certificate (Ref: presence)	1.000	0.985	**-0.120**		0.985	0.988	0.026	
46	Absence of severed body part code in the initial medical certificate (Ref: presence)	0.995	0.933	**-0.249**		0.940	0.938	-0.009	
47	Initial number of sick leave days prescribed	42.361	265.636	**1.127**	**4.602**	253.755	254.121	0.002	1.068
48	Number of sick leave days for vocational accident during the first month after the accident	18.673	30.353	**3.657**	**11.305**	30.476	30.488	0.005	1.024
49	Total amount of daily allowances for sick leave for vocational accident during the month after the accident	679.351	1,207.590	**0.802**	1.301	1,198.655	1,232.087	0.051	1.004
50	Consultations with general practitioners (healthcare reimbursements during the first month after the accident)	39.363	24.559	**-0.494**	1.307	25.348	24.185	-0.039	1.083
51	Nursing care (healthcare reimbursements during the first month after the accident)	9.007	80.331	**0.538**	**10.543**	72.913	72.218	-0.005	1.078
52	Physical therapy (healthcare reimbursements during the first month after the accident)	16.444	27.544	**0.197**	1.675	28.170	27.547	-0.011	1.303
53	Total healthcare benefits in kind from the vocational fund (except hospital care), during the first month after the accident	179.607	793.281	**0.520**	**12.070**	673.788	732.709	0.051	1.298
54	Total healthcare benefits in kind from the non-vocational sickness fund (except hospital care), during the first month after the accident	82.954	178.642	**0.250**	1.565	191.439	190.722	-0.002	1.562

N°	Variable	Before matching				After matching			
		Mean		Standardized bias	Variance Ratio	Mean		Standardized bias	Variance Ratio
		Control	Treated			Control	Treated		
55	Total healthcare benefits in cash from the vocational fund (except hospital care), during the first month after the accident	858.959	2,000.871	**0.822**	**3.532**	1,872.443	1,964.796	0.067	1.175
56	Total healthcare benefits in cash from the non-vocational sickness fund (except hospital care), during the first month after the accident	941.912	2,179.513	**0.836**	**3.253**	2,063.882	2,155.517	0.062	1.168
57	Patient not hospitalized during the first month after the accident (Ref: patient was hospitalized at least once)	0.925	0.368	**-1.152**		0.379	0.379	0.000	
58	Number of hospital stays during the first month after the accident	0.080	0.736	**0.980**	**5.417**	0.697	0.708	0.018	1.064
59	Length of stay at the hospital during the first month after the accident	0.150	3.810	**0.620**	**23.858**	3.422	3.675	0.044	1.027
60	No hospital stay imputed to the accident during the first month after the accident (Ref: At least one stay imputed)	0.950	0.494	**-0.910**		0.523	0.521	-0.005	
61	No diagnosis of traumatic injury for the first hospital stay during the first month after the accident (Ref: presence of such diagnosis)	0.944	0.394	**-1.124**		0.409	0.404	-0.010	
62	No surgery for the first hospital stay after the accident during the first month after the accident (Ref: Surgery)	0.952	0.465	**-0.974**		0.486	0.479	-0.013	
63	Number of medical acts during the first hospital stay after the accident during the first month after the accident	0.262	4.078	**0.736**	**17.521**	3.717	3.950	0.045	1.225

Notes:

a/ The difference between groups is considered significant if the absolute value of the standardized bias is above 0.1, and if the variance ratio - defined in cobalt such that the numerator is the greatest variance - is above 2.

Table OA.3: Descriptive statistics after matching on the smaller version of the control group

The smaller version of the control group included only injuries occurring in 2015 in the five insurance districts where the program was experimented

The values above the chosen thresholds of balance are indicated in bold [a/]

N°	Variable		Control	Treated	Standardized bias	Variance ratio
	Sample size		2,924	190		
1	Sex: Male (Ref: Female)		0.301	0.311	0.020	
2	Age		40.429	41.395	0.080	0.882
3	Number of beneficiaries of his/her social security coverage		1.613	1.547	-0.059	1.087
4	Size of the hometown	Rural	0.116	0.084	**-0.105**	
5		City up to 199 999 inhabitants	0.158	0.147	-0.030	
6		City of more than 200 000 inhabitants	0.459	0.505	0.092	
7		Greater Paris area	0.266	0.263	-0.007	
8	Deprivation index of the hometown	Most privileged quintile	0.261	0.268	0.016	
9		2nd quintile	0.281	0.263	-0.039	
10		3rd quintile	0.164	0.179	0.037	
11		4th quintile	0.124	0.105	-0.060	
12		Most deprived quintile	0.170	0.184	0.039	
13	Total previous permanent work incapacity		0.310	0.500	0.065	**2.970**
14	Number of previous occupational accidents		1.445	1.432	-0.007	1.031
15	Socio-professional category	Unspecified or miscellaneous	0.158	0.189	0.081	
16		Managers, technicians	0.079	0.063	-0.061	
17		Employees	0.348	0.316	-0.069	
18		Unskilled workers	0.133	0.158	0.071	
19		Skilled workers	0.283	0.274	-0.020	
20	Type of employment contract	Unknown or miscellaneous	0.238	0.247	0.021	

N°	Variable		Control	Treated	Standardized bias	Variance ratio
21		Permanent contract	0.649	0.621	-0.057	
22		Temporary contract	0.113	0.132	0.057	
23	Business sector of the firm	Commercial	0.357	0.337	-0.042	
24		Industrial	0.283	0.284	0.002	
25		Services and miscellaneous	0.360	0.379	0.040	
26	Size of the firm	Microenterprise (9 or less)	0.233	0.279	**0.102**	
27		Small and medium sized enterprise (10 to 249)	0.437	0.411	-0.053	
28		Intermediate size enterprise (250 to 4,999)	0.202	0.211	0.021	
29		Large company (5,000 or more)	0.128	0.100	-0.093	
30	Type of vocational accident: In the workplace (Ref: On the way to work)		0.183	0.237	**0.123**	
31	Type of injury	Imprecise	0.268	0.268	0.000	
32		Superficial injuries	0.011	0.011	0.000	
33		Open wounds	0.053	0.053	0.000	
34		Closed fractures	0.389	0.389	0.000	
35		Open fractures	0.074	0.074	0.000	
36		Dislocations, sprains and strains	0.163	0.163	0.000	
37		Traumatic amputations	0.042	0.042	0.000	
38	Location of the injury	Imprecise	0.116	0.116	0.000	
39		Neck, back and rib cage	0.132	0.132	0.000	
40		Upper limbs	0.347	0.347	0.000	
41		Lower limbs	0.405	0.405	0.000	
42	Number of eligible injury codes detected in the initial medical certificate		2.417	2.579	0.080	1.172
43	Absence of low back pain code in the initial medical certificate (Ref: presence)		0.145	0.147	0.006	
44	Absence of amputation code in the initial medical certificate (Ref: presence)		0.045	0.047	0.012	
45	Absence of multiple fractures code in the initial medical certificate (Ref: presence)		0.016	0.011	-0.053	
46	Absence of severed body part code in the initial medical certificate (Ref: presence)		0.046	0.058	0.052	

N°	Variable	Control	Treated	Standardized bias	Variance ratio
47	Initial number of sick leave days prescribed	232.181	231.226	-0.005	0.920
48	Number of sick leave days for vocational accident during the first month after the accident	30.424	30.432	0.003	1.014
49	Total amount of daily allowances for sick leave for occupational accident during the month after the accident	1,169.207	1,196.458	0.041	1.005
50	Consultations with general practitioners (healthcare reimbursements during the first month after the accident)	26.781	24.542	-0.075	0.793
51	Nursing care (healthcare reimbursements during the first month after the accident)	78.007	67.697	-0.078	0.796
52	Physical therapy (healthcare reimbursements during the first month after the accident)	29.265	31.664	0.042	0.786
53	Total healthcare benefits in kind from the vocational fund (except hospital care), during the first month after the accident	645.096	679.347	0.030	1.664
54	Total healthcare benefits in kind from the non-vocational sickness fund (except hospital care), during the first month after the accident	230.959	182.191	**-0.122**	**0.449**
55	Total healthcare benefits in cash from the vocational fund (except hospital care), during the first month after the accident	1,814.303	1,875.806	0.045	1.500
56	Total healthcare benefits in cash from the non-vocational sickness fund (except hospital care), during the first month after the accident	2,045.262	2,057.996	0.009	1.370
57	Patient not hospitalized during the first month after the accident (Ref: patient was hospitalized at least once)	0.563	0.563	0.000	
58	Number of hospital stays during the first month after the accident	0.614	0.642	0.043	1.254
59	Length of stay at the hospital during the first month after the accident	2.761	3.189	0.073	0.888
60	No hospital stay imputed to the accident during the first month after the accident (Ref: At least one stay imputed)	0.425	0.416	-0.018	
61	No diagnosis of traumatic injury for the first hospital stay during the first month after the accident (Ref: presence of such diagnosis)	0.534	0.532	-0.006	

N°	Variable	Control	Treated	Standardized bias	Variance ratio
62	No surgery for the first hospital stay after the accident during the first month after the accident (Ref: Surgery)	0.462	0.463	0.002	
63	Number of medical acts during the first hospital stay after the accident during the first month after the accident	3.499	3.542	0.008	1.023

Notes:

a/ The difference between groups is considered significant if the absolute value of the standardized bias is above 0.1, and if the variance ratio - defined in cobalt such that the numerator is the greatest variance - is above 2

Table OA.4: Average Treatment effects on the Treated (ATT) after matching on the smaller version of the control group

The smaller version of the control group included only injuries occurring in 2015 in the five insurance districts where the program was experimented

N°	Outcome variable	Control Group (weighted)[a/]	Treatment Group (weighted)[a/]	ATT [confidence intervals][b/]	Standard Errors	P-value	Ratio treated / control group [confidence intervals][c/]
1	Vocational sick leave days	223.694	251.916	29.178*** [18.797 - 39.559]	5.294	<0.001	1.1 [1.0-1.1]
2	Non-vocational sick leave days	7.967	3.563	-4.235* [-9.205 - 0.736]	2.535	0.095	0.4 [-0.-1.0]
3	Part-time RTW	10.465	14.574	4.452* [-0.716 - 9.621]	2.636	0.091	1.4 [0.9-1.9]
4	Workers with final medical certificate	0.537	0.505	-0.021 [-0.076 - 0.034]	0.028	0.45	0.9 [0.8-1.0]
5	Workers with a permanent work incapacity (IP>0)	0.063	0.232	0.168*** [0.135 - 0.202]	0.017	<0.001	3.6 [3.1-4.2]
6	Workers with a severe permanent work incapacity (IP>9)	0.003	0.032	0.027*** [0.018 - 0.036]	0.005	<0.001	10 [7-13]
7	Level of permanent work incapacity	0.306	1.426	1.11*** [0.906 - 1.313]	0.104	<0.001	4.6 [3.9-5.2]
8	Daily allowances for vocational sick leave (in euros)	10,088.147	11,772.1	1,727.702*** [1,067.172 - 2,388.231]	336.878	<0.001	1.1 [1.1-1.2]
9	One-off indemnities (in euros)	119.667	463.19	341.545*** [264.132 - 418.957]	39.481	<0.001	3.8 [3.2-4.5]
10	Life-long disability pensions (in euros)	28.961	95.356	48.792*** [12.044 - 85.540]	18.742	0.009	2.6 [1.4-3.9]

N°	Outcome variable	Control Group (weighted)[a/]	Treatment Group (weighted)[a/]	ATT [confidence intervals][b/]	Standard Errors	P-value	Ratio treated / control group [confidence intervals][c/]
11	Healthcare costs for vocational injuries (in euros)	2,512.954	3,558.406	975.082*** [646.505 – 1,303.660]	167.579	<0.001	1.3 [1.2-1.5]
12	Healthcare costs for non-vocational sickness (in euros)	1,036.76	1,083.421	45.9 [-214.872 - 306.671]	132.997	0.73	1.0 [0.7-1.2]
13	Total benefits (in euros)	14,006.666	17,086.011	3,051.972*** [2,206.452 – 3,897.493]	431.225	<0.001	1.2 [1.1-1.2]

a/ Sample sizes: control group N= 190 and treatment group N= 2,924.

b/ Calculated using a linear regression model in the matched sample using all the covariates as controls. Significance of coefficients set at * p-value 10% level, ** p-value 5% level, and *** p-value 1% level.

c/ Ratio calculated using the ATT, as the sum of the control group value (1) + ATT value (2) divided by the control group value (1): (1+2)/(1). Confidence intervals calculated as the sum of the control group value (1) + lower (upper) bound of ATT's confidence interval (2) divided by the control group value (1): (1+2)/(1).
